# Chronic disease risk factors associated with health service use in the elderly

**DOI:** 10.1186/1472-6963-8-237

**Published:** 2008-11-15

**Authors:** Sarah Maaten, George Kephart, Susan Kirkland, Pantelis Andreou

**Affiliations:** 1 Elgin St. Thomas Public Health, 99 Edward St., St Thomas, Ontario N5P 1Y8, Canada; 2 Department of Community Health & Epidemiology, Dalhousie University, Halifax, Nova Scotia, Canada

## Abstract

**Background:**

To examine the association between number and combination of chronic disease risk factors on health service use.

**Methods:**

Data from the 1995 Nova Scotia Health Survey (n = 2,653) was linked to provincial health services administrative databases. Multivariate regression models were developed that included important interactions between risk factors and were stratified by sex and at age 50. Negative-binomial regression models were estimated using generalized estimating equations assuming an autoregressive covariance structure.

**Results:**

As the number of chronic disease risk factors increased so did the number of annual general practitioner visits, specialist visits and days spent in hospital in people aged 50 and older. This was not seen among individuals under age 50. Comparison of smokers, people with high blood pressure and people with high cholesterol showed no significantly different impact on health service use.

**Conclusion:**

As the number of chronic disease risk factors increased so did health service use among individuals over age 50 but risk factor combination had no impact.

## Background

Chronic diseases are responsible for the majority of deaths worldwide [1]. The baby-boomer generation makes up a large portion of the North American population, and there will likely be an increase in absolute numbers of heart attacks and strokes as this group reaches the age traditionally associated with the onset of cardiovascular disease, even if the rates of these events decline [2-4]. Cardiovascular disease is not the only concern: other chronic diseases such as cancer, pulmonary diseases, diabetes and osteoporosis are likely to increase in number [5,6]. Risk factors such as smoking, high blood pressure, cholesterol, obesity, physical inactivity and diabetes are common to many of these chronic diseases. Primary prevention of even a few of these factors could result in an increase in disability-free years and ultimately, a reduction in health care costs [7-9].

Quantifying the impact of chronic disease risk factors on health care use can be used to estimate the return on investment of health promotion and other policies designed to prevent chronic disease. It also provides a powerful means of communicating the value of risk factor modification to the public and policy makers. However, estimating the effect of chronic disease risk factors on health service use is complex, and previous studies have been subject to some important limitations. In the absence of long-term longitudinal data to support a life course perspective, measuring the effects of risk factors within cohorts is difficult. There are tradeoffs between modelling the complexity of how risk factors interact, on the one hand, and conveying the results in a clear fashion on the other hand. As a result, risk factor measurement is often oversimplified, relying on the reporting of main effects only rather than incorporating higher order interactions to facilitate ease of interpretation. Such oversimplifications may bias results and underestimate the true impact of chronic disease risk factors on a variety of outcomes such as morbidity, mortality and health service use.

The aim of this study was to measure the effect of chronic disease risk factors on health services utilization in Nova Scotia, Canada. The first objective of this study was to estimate the effects of number and combination of risk factors on health service utilization, as measured by family physician visits, specialist physician visits, and inpatient hospital bed days in different age groups of women and men. A second objective was to assess the relative importance of three major risk factors – smoking, high blood pressure and high cholesterol, on health care utilization.

The approach we utilized overcomes the limitations noted by separating the task of modelling the relationship between risk factors and health services use from the task of summarizing, for communication purposes, variation in health services use by risk factor combination. This allows for a complicated modelling strategy to better describe the relationship between risk factor and service use with an accessible presentation of the results.

## Methods

### Overview of study design

A retrospective cohort study was conducted. Participants from the 1995 Nova Scotia Health Survey (NSHS) with information on risk factors were linked to provincial health services utilization data and followed prospectively for six years. Individuals were assessed based on their 1995 exposure to major risk factors for chronic disease. The outcome under study was health service utilization, as measured by the average annual number of GP visits, specialist visits and hospital bed days between the NSHS interview date in 1995 and March 31, 2001. The outcomes were compared for groups with varying numbers and combinations of risk factors.

### Sample

The 1995 Nova Scotia Health Survey was a population-based survey designed to provide a cross sectional picture of the health status, risk factors and preventive health practices of Nova Scotians [10]. In total, 5578 non-institutionalized adults from all parts of the province were drawn from the provincial Medical Services Insurance database, a comprehensive list of all Nova Scotians eligible to use heath services. Canada has a comprehensive medical insurance system that provides all necessary medical services to all Canadians; as a result, the list of insured participants is considered to be a comprehensive registry of the population. From this sampling, 4360 people were located and 3227 participated. The current study was comprised of the 82% of survey respondents (n = 2658) who completed a clinic visit where blood pressure, BMI and blood cholesterol measurements were taken. Over 99% of all clinic respondents gave consent (n = 2653) to link their survey information with administrative health records. Weights applied to adjust for those we were unable to locate and propensity score weights adjusting for non-response showed no meaningful biases in cardiovascular risk factors among clinic participants compared to the general population [11]. A detailed methodology of the NSHS is described elsewhere [12].

### Data

The NSHS data was linked to provincial administrative health databases containing information on insured health services. Information was linked through the use of encrypted identification numbers to ensure confidentiality; no identifying information was available to the investigators. The study received ethics review and approval by the Dalhousie University Health Sciences Human Research Ethics Board.

Two sources of health service information were utilized: the Hospital Discharge Abstract Database; and, the Medical Services Insurance (MSI) Physician Services File. The MSI Registry file was used to identify participants who died or migrated out of the province during the study period. Annual Hospital Bed Days were summed; individual hospital admissions with a length of stay longer than 60 days were excluded to eliminate hospitalizations that reflected patterns more similar to long term care. The MSI Physician Services File contained fee-for-service doctor visits, both to general practitioners and specialists. The number of visits by each participant each year was extracted. Chronic disease permeates several aspects of health service utilization and can be implicated in many diagnoses; therefore, all services for all ICD-9-CM diagnostic codes were included. This likely provided an overrepresentation of chronic disease health service use, however, the group with no risk factors provided baseline health service utilization unrelated to chronic disease against which all other risk factor profiles were compared.

### Measures

Continuous measurement of variables such as blood pressure, cholesterol and obesity, annual GP visits, annual specialist visits and hospital bed days was used to enhance the ability of the statistical models to estimate the variance in utilization attributable to risk factors. Many of the variables in previous risk factor studies were aggregated into a few discrete categories rather than being left on an interval scale with many gradations. Unfortunately, categorization sacrifices the ability to measure the variation that is seen within each level of the variable. This variability may be crucial to predict service use or mortality outcomes precisely.

### Statistical Analysis

#### Descriptive Statistics

Descriptive statistics were calculated to characterize the distribution of risk factors in the sample population. Mean, median and percentage values were calculated.

#### Complex Modelling

The models employed person-years as the unit of analysis to measure the association between risk factors and health service utilization. Regressions models were estimated using generalized estimating equations (GEE); a first order autoregressive correlation matrix for errors was specified for the GEE procedure. The negative binomial distribution was selected for all outcomes, based on its fit with the observed distribution of health services use. The study participants were stratified into four categories by sex and age (dichotomized at 50 years). Final models were built for the 12 groups (3 outcomes × 4 age-sex strata) to best predict health service utilization in each stratum.

We engaged in a process to build models that were parsimonious, yet sufficiently complex to capture the interactions between risk factors, and between risk factors and age. Initially, each of the 12 models was run with only main effects for risk factors, age and the socio-demographic variables. The relationship between the risk factors and outcomes is likely to vary with age, so interaction terms for each risk factor and age were included in the model. A number of two-way and three-way risk factor interaction terms, derived from previous reports of statistically significant interactions in chronic disease outcomes found in the literature, were also included [13-18]. Each interaction term was assessed for its contribution to the model based on the difference in Wald Chi-square statistic using a minimum significance level of 0.05.

A set of regression models that described utilization as a function of risk factor combinations and age for each age-sex stratum was developed in the modelling phase. The final models included two and three-way interaction terms between categorical and continuous variables. These models were different for each age-sex strata.

#### Results Interpretation and Summary

While the complex regression models are well suited for modelling variation in utilization associated with risk factors, they cannot be easily interpreted. The final step in the analysis focused on summarizing the results of the models in a meaningful way. An important methodological contribution of this study shows that modelling need not be compromised to facilitate the communication of results.

All of the observations were divided into age and sex strata and the corresponding model developed for each stratum was estimated using the appropriate data. Predicted utilization estimates were obtained for each person-year observation and then summarized to obtain the mean estimated (predicted) health service use for patients with different numbers and combinations of risk factors.

Possible confounding variables were held constant within each of the 12 strata to estimate only the effect of risk factors. These variables (age, geographic location, education, employment status and exposure) were held constant at the mean value for each stratum.

For summary purposes, each participant was re-classified as high or low risk for each risk factor. The following cut points were used to designate study participants as high or low in risk status:

• Hypertensive – mean systolic >= 140 mmHg or mean diastolic >= 90 mmHg.

• High Cholesterol – total cholesterol >= 5.2 mmol/L

• Smoker – currently smokes or quit <= 5 years prior to survey

• Obesity – BMI >= 27

• Diabetes – Self-identified as having Type I or II diabetes and reported being seen by a medical professional about diabetes treatment

• Physical Inactivity – Exercised less than three times per week.

Each person year observation was evaluated based on the cut points outlined above and the predicted values were then averaged to obtain the mean number of annual doctor visits or annual hospital bed days for those individuals with 0, 1, 2, 3, and 4 + risk factors. Means were weighted to adjust for the complex sample design. Because age plays such a large role in the severity of chronic disease and level of service utilization, predicted values were generated holding age constant at 25, 33.3 (average age of under 50 cohort), 45, 55, 65.7 (average age of 50 & over cohort) & 75. Predicted data was not stratified by sex because similar utilization by risk factor combination was found between men and women.

Individuals with 1, 2 and 3 risk factors were further stratified to assess which of the major risk factors traditionally examined in the cardiovascular and chronic disease literature (high blood pressure, high cholesterol, smoking) had the greatest impact on service utilization. This was done holding age constant only at 33.7 and 65.7 (the mean ages of the old and young strata).

### Software

Data preparation was conducted using SAS Version 8. Descriptive statistics and the full analysis were conducted using STATA Version 8.

## Results

### Participant characteristics

There were a total of 13676 person-years representing 2523 individuals included in all regressions. 130 individuals were removed from regressions due to missing data. Large differences were seen in prevalence of risk factors between age/sex strata, indicating that it was valuable to stratify by sex and at age 50 at the modelling stage (Table 1). Large variations in risk factor status and health service utilization were seen between men and women as well as between the young and old

**Table 1:** Mean Baseline Characteristics of Study Participants Included in the Regressions

**Characteristic**	**Men 50 and Over** **(N = 557)**	**Men Under 50** **(N = 693)**	**Women 50 and Over** **(N = 588)**	**Women Under 50** **(N = 685)**
	%			

Current smoker	20.7	36.3	18.1	32.8
Physically Inactive	40.7	45.8	45.6	40.9
Diabetic	6.1	1.0	6.4	1.0
HS diploma or more	48.7	79.7	52.5	83.7
Geographic Location				
Central	29.8	32.2	32.5	35.0
Northern	26.6	25.9	24.15	22.4
Eastern	23.1	22.7	20.6	21.0
Western	20.5	19.2	22.7	21.6
Unemployed in current year	7.0	29.2	6.2	26.4

	Mean (SD) [Median]			

Age in years	66.5 (10.3)	33.0 (9.6)	66.2 (10.4)	33.1 (9.2)
Total Cholesterol in mmol/L	5.5 (1.0)	5.1 (1.1)	5.9 (1.1)	4.9 (0.9)
Diastolic Blood Pressure in mm Hg	79.8 (9.3)	78.4 (9.6)	77.3 (8.5)	72.7 (8.6)
Systolic Blood Pressure in mm Hg	136.0 (17.6)	122.5 (12.0)	133.9 (17.9)	112.8 (12.1)
Body Mass Index	27.4 (4.4)	27.2 (5.4)	27.3 (5.7)	26.2 (6.2)
Annual GP visits	7.0 (9.1) [5]	2.8 (4.4) [1]	7.7 (8.8) [6]	5.1 (5.5) [4]
Annual Specialist visits	4.4 (8.8) [1]	1.2 (3.8) [0]	3.7 (6.0) [1]	1.7 (3.9) [0]
Annual hospital bed days	2.6 (7.9) [0]	1.1 (4.8) [0]	2.1 (6.9) [0]	1.0 (4.0) [0]

SD = standard deviation

*Results are not weighted. Numbers represent only the study sample.

### Regression Models

Models were constructed for each outcome: number of annual GP visits, number of annual specialist visits, and number of annual hospital bed-days. The data indicated that risk factor synergies, whether they were interacting with other risk factors or age were not particularly important in predicting GP use. Age/risk interactions were more important than risk/risk interactions in predicting specialist visits but hospital visits were affected by both age/risk interactions and risk/risk interactions.

### The association between number of risk factors and utilization

The number of chronic disease risk factors had an impact on health service use for individuals over the age 50, but not for those under age 50 (Table 2). This was true regardless of the type of health service use examined. Therefore, we focus our results with respect to number of risk factors on those over 50.

**Table 2:** Predicted Mean Annual GP Visits, Specialist Visits and Hospital Bed Days by Different Risk Number Profiles Considering All 6 Risk Factors for Individuals 50 & Older

**Number of Risk Factors**		**0**	**1**	**2**	**3**	**4+**
*Sample Size*		*50*	*267*	*377*	*291*	*212*

						

Mean number of GP Visits (95% CI)	AGE 55	5.67 (5.37, 5.97)	5.85 (5.74, 5.97)	6.37 (6.27, 6.47)	6.69 (6.56, 6.82)	7.38 (7.18, 7.59)
	
	AGE 65.7	7.17 (6.92, 7.42)	7.36 (7.24, 7.48)	7.95 (7.84, 8.06)	8.45 (8.31, 8.58)	9.31 (9.05, 9.56)
	
	AGE 75	8.83 (8.62, 9.04)	9.02 (8.88, 9.17)	9.69 (9.54, 9.84)	10.39 (10.20, 10.57)	11.43 (11.10, 11.77)

Mean number of Specialist Visits (95% CI)	AGE 55	3.13 (2.89, 3.38)	3.01 (2.86, 3.16)	2.90 (2.75, 3.04)	2.93 (2.75, 3.12)	2.91 (2.65, 3.16)
	
	AGE 65.7	4.37 (4.12, 4.63)	4.40 (4.21, 4.58)	4.46 (4.26, 4.66)	4.71 (4.44, 4.99)	4.91 (4.50, 5.32)
	
	AGE 75	5.07 (4.82, 5.33)	5.32 (5.11, 5.53)	5.70 (5.43, 5.96)	6.32 (5.93, 6.71)	7.09 (6.43, 7.75)

Mean number of Hospital Bed Days (95% CI)	AGE 55	1.42 (1.14, 1.70)	1.47 (1.32, 1.62)	1.28 (1.16, 1.40)	0.99 (0.91, 1.08)	0.92 (0.79, 1.05)
	
	AGE 65.7	1.69 (1.40, 1.97)	1.82 (1.66, 1.98)	1.84 (1.69, 2.00)	1.93 (1.76, 2.10)	2.29 (2.06, 2.52)
	
	AGE 75	2.10 (1.75, 2.45)	2.85 (2.32, 3.37)	3.57 (2.96, 4.18)	4.83 (3.91, 5.74)	7.43 (6.08, 8.77)

* Holding all study participants' age constant at the age specified, education, employment status and region constant at the sample proportions.

Survey weights were applied to get weighted means and corrected standard errors

**Figure 1:**
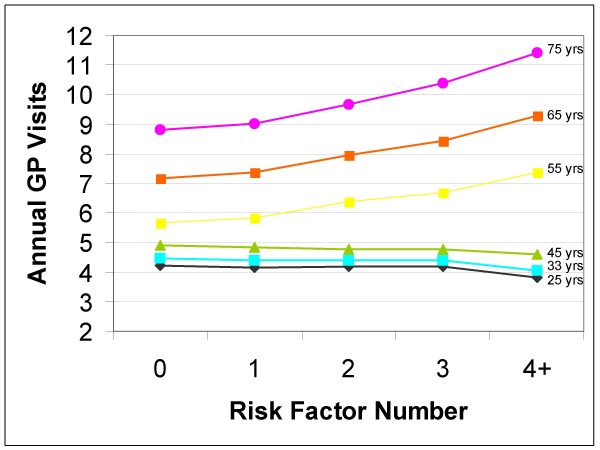
Average Annual Predicted GP Visits by Age.

There was a consistent upward trend in use as the number of risk factors increased at age 55, 65 and 75 for GP visits and after age 65 for hospital and specialist use (Figure 1, Table 2). For example, individuals aged 55 with zero risk factors had an average of 5.67 (95%CI 5.37, 5.97) annual GP visits compared to 7.38 (95%CI 7.18, 7.59) annual visits in 55 year olds with four or more risk factors.

**Figure 2:**
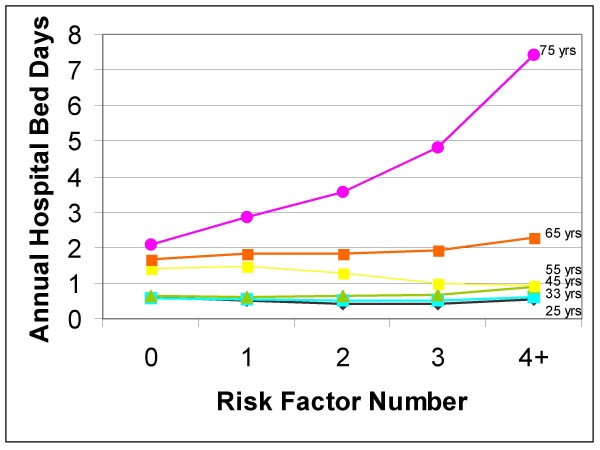
Average Annual Predicted Hospital Bed Days by Age.

The effect of risk factors on health service use in the '50 and over' population varied by age for hospital bed days and specialist visits but not for GP visits (Figure 1, Table 2). The slopes of the graphs of annual GP use at ages 55, 65 and 75 were similar indicating a proportionate rise in use of services regardless of age (Figure 1). Specialist visits increased disproportionately as the number of risks increased. There was a slight increase in use for those over age 65 [from 4.37 (95%CI 4.12, 4.63) to 4.91 (95%CI 4.50, 5.32)] but a more pronounced increase at age 75 with more risk factors [from 5.07 (95%CI 4.82, 5.33) to 7.09 (95%CI 6.43, 7.75)]. The increase in hospital use with increasing age was the most dramatic of all types of health service examined. For example, average annual hospital bed days in those 75 years old differed dramatically between those with no risk factors [2.10 (95%CI 1.75, 2.45)] and those with four or more risk factors [7.43 (95%CI 6.08, 8.77)]. The hospital use rates at age 75 are dramatic; however there does not appear to be much influence of risk factors on hospital use until age 75 (Figure 2, Table 2).

#### Type of Risk Factors

A comparison of the average number of doctor visits and hospital bed days for people with high blood pressure, high cholesterol or smoking in their risk profile showed that the specific combination of risk factors had little effect on service use (Table 3). Smoking, high blood pressure and high cholesterol status also had no effect on health service use in individuals less than 50 years. This result is not surprising since the number of risk factors did not predict health service use in individuals less than 50 years.

**Table 3:** Predicted Mean Annual GP Visits, Specialist Visits and Hospital Bed Days by Different Risk Profiles Considering only Smoking, High Blood Pressure and High Cholesterol in Individuals 50 & Older

**No. of Risk ** **Factors**	**Combination**	**Sample Size**	**Mean number of ** **GP Visits** **(95% CI)**	**Mean number of ** **Hospital Bed Days** **(95% CI)**	**Mean number of ** **Specialist Visits** **(95% CI)**
*None*	None	50	7.17 (6.92, 7.42)	1.69 (1.40, 1.97)	4.37 (4.12, 4.63)

*1 Risk Factor*	Any One	267	7.36 (7.24, 7.48)	1.82 (1.66, 1.98)	4.40 (4.21, 4.58)
	
	High Blood Pressure	27	7.08 (6.69, 7.48)	1.98 (1.58, 2.38)	4.54 (4.28, 4.79)
	
	Smoking	21	7.24 (6.75, 7.74)	1.17 (0.82, 1.53)	3.83 (2.86, 4.79)
	
	High Cholesterol	147	6.96 (6.86, 7.06)	1.32 (1.22, 1.43)	3.76 (3.66, 3.86)

*2 Risk Factors*	Any Two	377	7.95 (7.84, 8.06)	1.84 (1.69, 2.00)	4.46 (4.26, 4.66)
	
	High Blood Pressure + 1 Other	123	7.61 (7.42, 7.80)	1.82 (1.65, 2.00)	4.45 (4.19, 4.71)
	
	Smoking + 1 Other	71	7.80 (7.60, 8.01)	1.45 (1.23, 1.67)	3.28 (2.55, 4.01)
	
	High Cholesterol + 1 Other	261	7.63 (7.52, 7.74)	1.57 (1.43, 1.70)	3.99 (3.80, 4.18)

*3 Risk Factors*	Any Three	291	8.45 (8.31, 8.58)	1.93 (1.76, 2.10)	4.71 (4.44, 4.99)
	
	High Blood Pressure + 2 Others	141	8.37 (8.15, 8.60)	1.90 (1.68, 2.13)	4.81 (4.46, 5.17)
	
	Smoking + 2 Others	106	8.36 (8.13, 8.59)	1.71 (1.49, 1.94)	3.96 (3.33, 4.59)
	
	High Cholesterol + 2 Others	238	8.26 (8.13, 8.39)	1.80 (1.63, 1.97)	4.50 (4.23, 4.76)

*This was done holding all study participants' age constant at the stratum mean (65.7), education, employment status and region constant at the sample proportions.

Survey weights were applied to get weighted means and corrected standard errors

## Discussion

Health service utilization was found to be dependent on the number of chronic disease risk factors only among individuals over the age of 50. People over the age of 50 with many risks factors (4+) went to the GP almost 30% more often than those with no risk factors; a similar result was found in annual specialist visits and hospital use. The effect of number of risk factors on specialist and hospital use was not seen until age 65. After age 75 the high-risk group was found to have 40% higher specialist use and 350% higher hospital visits than the group with no risk factors.

The finding that hospital use increased as the number of risk factors increased in older populations is consistent with previous work. Daviglus et al, found that costs related to hospital use increased significantly when comparing individuals with some risk to those with no risk [19]. Our sub-analysis by smoking, high blood pressure and cholesterol status indicated that the specific combination of risk factors was not important in predicting health service use. These results differed from Daviglus et al. who found that high blood pressure was less costly than smoking and high cholesterol. The differing results may be due to our inclusion of synergistic relationships between risk factors. Our method of separating service use modelling from results presentation may have allowed us to more accurately measure the impact of multiple risk factors present in an individual. Our approach suggests that it is the number of risks and not the type of risks that impact service use.

Examination of the effect of risk factors on health service use under the age of 50 revealed that the number of risk factors had no effect on the use of any type of health service. However, chronic disease manifests late in life after many years of exposure to risk factors. Due to this threshold effect, those with the highest risk profiles at younger ages are probably no more likely to have overt CVD, cancer or osteoporosis than those with no risks. It is, therefore, not surprising that young people with many risks had similar use to those with no risks.

Surprisingly it was found that, for individuals under age 50, annual GP visits, specialist visits and, to some degree, hospital bed days decreased slightly as the number of risks increased. Regular visits to the GP often include messages of healthy lifestyle and primary prevention of disease. Our data could support two competing possibilities: more frequent visits to the GP encourage the development of fewer risk factors, or those with more risk factors are more likely to avoid their general practitioner. The models developed in this study cannot distinguish between these two explanations, and serves to highlight the possible endogenous relationship between risk factors and health service use that requires further exploration in future studies.

Modelling three outcomes in two different sexes in two different age groups provided the opportunity to examine the risk factors that impact health service use in different age/sex groups of the population. The synergy of risk factors appeared to have the greatest effect on hospital use. That is, the largest number of risk factor interaction terms remained significant in the hospital use model compared to the other health services. Particularly in those aged 50 and older, many age by risk factor combinations as well as combinations of different risk factors had a significant association with hospital use. This was also seen, to a lesser degree, in hospital use in the younger age strata. Synergistic effects of risk factors by age and risk factor by risk factor also appeared to have an effect on specialist visits, much more so than general practitioner visits. In fact, there was no significant interaction term predicting general practitioner use in women aged 50 and older. It is not a surprising finding that hospital visits were more likely to be affected by risk factor interactions than doctor visits. GP visits are often routine check-ups and are just as likely to represent primary prevention of disease as opposed to treatment of disease. One is more likely to be afflicted with a chronic disease if they are hospitalized compared with if they are visiting the general practitioner.

Limitations from this study may have arisen from the cross-sectional nature of the survey data and the self-report of the variables. Measurement error may have been introduced with the self-reported variables such as diabetes [20]. However, the self-report limitation was not a factor in the measurement of BMI, cholesterol levels and blood pressure. These factors were measured in the clinic portion of the 95NSHS, which increased the accuracy of measurement of the level of risk in participants. Health services administrative data is more reliable source of utilization than self-reported service use [20].

It is important to note that standard errors and confidence intervals presented in the tables represent only the error from the variation between summarized predicted values. The independent variable coefficients or point estimates for each independent risk factor also include a standard error of the estimate, as do the mean values of systolic and diastolic blood pressure. The modelling/summarization approach allows for sophisticated modelling of interaction terms but sacrifices the ability to assess the additive effects of standard errors at multiple levels. Thus, confidence intervals and p-values were likely underestimated in the summarized results, and may not be appropriate for determining statistically significant differences between different numbers or combinations or risk factors.

## Conclusion

The complex measurement and modelling presented in this study provides an example of how the complicated web of risk factors can be more accurately modelled in relation to health service use. Inclusion of six major risk factors for chronic diseases of the cardiovascular, respiratory, musculo-skeletal systems and cancer allows us to make broad inferences about the effect of chronic disease risk factors on health service utilization. The number of chronic disease risk factors had a big impact on health service utilization after the age of 50. As hypothesized, the number of chronic disease risk factors was a better predictor of health service use than the combination of risk factors. Smoking, high blood pressure and high cholesterol had equal effects on all types of service use at all ages. This result differs from previous work perhaps because of the increased precision of modelling risk factors level and risk factor synergies.

These results suggest that primary prevention of chronic disease risk factors should be conducted at a population level if we are ever to hope to reduce consumption of health service use. Although rates of risk factors, such as smoking, have decreased in recent years in young people there are still relatively high rates in the young adult population [21]. These will need to be addressed in order to stem the increase of chronic disease in the future. Given the age of the data, more than 10 years have passed since the time of the 95NSHS and chronic disease rates have been rising in that time frame. Thus, the impact on health service use today is a conservative estimate, and is likely to be greater than is reflected in these data.

Central to improving the health of the population is assessment of our government policy initiatives for their impact on health. There is a need to develop long-term health plans that will be unaffected by budget cycles and high turnover rates. Policy changes are needed to reduce the prevalence of chronic disease rather than placing the responsibility solely on the individual.

## Competing interests

The authors declare that they have no competing interests.

## Authors' contributions

SM has made substantive intellectual contributions to the conception and design, carried out acquisition of data, analysis and interpretation of data, has been involved in drafting the manuscript and has given final approval of the version to be published.

GK has made substantive intellectual contributions to the conception and design, has been involved in revising the manuscript critically for important intellectual content and has given final approval of the version to be published.

SK has made substantive intellectual contributions to the conception and design, has been involved in revising the manuscript critically for important intellectual content and has given final approval of the version to be published.

PA has made substantive intellectual contributions to the conception and design, has been involved in revising the manuscript critically for important intellectual content and has given final approval of the version to be published.

## Pre-publication history

The pre-publication history for this paper can be accessed here:


